# Lessons learnt from managing a case of dengue hemorrhagic fever complicated with acute liver failure and acute kidney injury: a case report

**DOI:** 10.1186/s13256-018-1766-0

**Published:** 2018-08-08

**Authors:** Chamara Dalugama, Indika Bandara Gawarammana

**Affiliations:** Department of Medicine, University of Peradeniya, Peradeniya, Sri Lanka

**Keywords:** Dengue hemorrhagic fever, Liver failure, Acute kidney injury, N-acetyl cysteine, Packed cell transfusion, Continuous veno-venous hemodialysis (CVVHD)

## Abstract

**Background:**

Dengue is a common arboviral infection with a diverse spectrum of clinical manifestations. Dengue hemorrhagic fever is a more severe form of infection characterized by plasma leak and hemoconcentration. Although hepatic dysfunction is common in dengue illness, massive liver necrosis is rarely reported. Lactic acidosis is a poor prognostic marker in liver failure related to dengue. Management of acute renal injury in dengue hemorrhagic fever due to prolonged shock is challenging as the fluid reabsorption during the recovery phase expands the intravascular volume and precipitates heart failure and pulmonary edema.

**Case presentation:**

We report the case of a 43-year-old Sri Lankan Sinhalese woman with serologically confirmed dengue fever presenting with evidence of plasma leakage developing acute liver failure evidenced by deranged liver functions, coagulopathy, and altered sensorium and acute kidney injury with anuria. She had elevated serum lactate levels. In addition to the “standard care,” she was managed with intravenously administered N-acetyl cysteine and blood transfusions, even in the absence of bleeding or dropping packed cell volume, targeting a higher packed cell volume anticipating a better oxygenation at tissue level. Continuous veno-venous hemodialysis was employed and continued for 138 hours removing the fluids reabsorbed during the recovery phase to prevent her from developing heart failure and pulmonary edema. She made full recovery with no sequelae.

**Conclusions:**

N-acetyl cysteine and packed cell transfusion aiming at a higher packed cell volume to maintain adequate tissue perfusion during shock may be beneficial in acute liver failure due to dengue virus. The use of a continuous form of renal replacement such as continuous veno-venous hemodialysis is of paramount importance in managing fluid states in the recovery phase of dengue hemorrhagic fever in those with renal impairment. Interesting observations made in the fluid dynamics during the reabsorption phase need further studies preferably with an animal model.

## Background

Dengue fever (DF) is a common arboviral disease that is endemic in Southeast Asia; DF has four distinct serotypes [1]. Dengue illness has a diverse clinical spectrum ranging from asymptomatic subclinical infection to severe multiorgan involvement and death [2]. Dengue illness encompasses changeable unusual manifestations [3–5]. Hepatic dysfunction is well reported both in DF and dengue hemorrhagic fever (DHF). Liver involvement can be varied ranging from mild to moderate elevation of serum transaminases to fulminant liver failure [6–13]. Various mechanisms are postulated to explain the hepatic dysfunction seen in dengue illness including direct viral damage, immunological injury, and hypoxic injury due to reduced hepatic perfusion during shock [14–21]. The role of N-acetyl cysteine (NAC) in liver injury is debated [22–24]. Lactic acidosis is a poor prognostic marker in DHF [25–32]. Acute oliguric renal failure is known to occur in patients presenting with prolonged shock. Renal impairment with oliguria raises concerns because the reabsorption of fluid during the recovery phase of DHF can lead to fluid overload and pulmonary edema. We report a case of a late presentation of DHF complicated with liver necrosis, lactic acidosis, and acute renal failure managed successfully. The use of NAC, the use of serum lactate levels to monitor improvement, the role of pack cell transfusion to improve tissue level oxygenation, and prophylactic employment of continuous veno-venous hemodialysis (CVVHD) anticipating the fluid overload during recovery in the background of renal impairment are discussed as important management strategies. Interesting observations on fluid dynamics were made during the fluid reabsorption in the recovery phase.

## Case presentation

We report a case of a 43-year-old Sri Lankan Sinhalese woman who presented to our Teaching Hospital, Peradeniya, in the morning with a history of fever, arthralgia, myalgia, and headache of 4 days’ duration. She had been previously diagnosed as having type 2 diabetes mellitus and dyslipidemia for which she received treatment with satisfactory control of the medical conditions and she had normal renal functions.

She had experienced postural dizziness since the afternoon of the previous day and she had had nausea, vomiting, and abdominal pain since the morning of the previous day. Soon after admission she collapsed in our emergency care unit. She was severely dehydrated with cold clammy peripheries. Her pulse rate was 130 beats per minute with an unrecordable blood pressure. She had reduced breath sounds in the base of her right lung and marked tenderness over the right hypochondrium with flank dullness with shifting. She was drowsy, but arousable.

Her complete blood count showed hemoglobin of 15.3 g/dL, platelet count of 74 × 10^3^/microL, and white cell count of 3.22 × 10^6^/microL. Her serum creatinine was 277 micromole/L with potassium of 5.8 mmol/L. Alanine transaminase (ALT) was 6542 U/L with aspartate transaminase (AST) of 30,617 U/L. Her serum albumin value was 24 g/L. A bedside ultrasound scan demonstrated bilateral pleural effusions (more in the right) with free fluid in her abdomen. A diagnosis of DHF with decompensated shock complicated with acute liver failure and acute kidney injury was made. DF was confirmed by positive non-structural protein 1 (NS1) antigen and serotype was identified as dengue virus type 2 (DEN-2). Both dengue immunoglobulin M (IgM) and immunoglobulin G (IgG) were positive suggesting a secondary infection with dengue virus (DENV). She was screened for alternative causes for liver necrosis including hepatitis A immunoglobulin A, hepatitis B surface antigen, hepatitis C IgM, leptospirosis serology, and rickettsial serology, which were negative. Her baseline transaminase levels and serum creatinine done 1 month earlier at a medical clinic were within the normal range. She denied taking supra-therapeutic dose of paracetamol or other native medical preparations for her fever. She had been treated with metformin and atorvastatin until the previous night.

She was assumed to be at the peak of the critical phase of DF (that is, 24 hours in the plasma leakage) on admission. On admission her packed cell volume (PCV) was 44%. (Her baseline PCV was 33% for hemoglobin of 10.5 g/dL in her clinic book.) She was given a 10 ml/kg crystalloid bolus (500 ml) over 15 minutes followed by 10 ml/kg bolus over 1 hour. She was given a 10 ml/kg dextran 40% bolus in the next hour. She had heavy per vaginal bleeding and one episode of melena. Her PCV dropped from 44 to 33% without clinical improvement and she had low urine output (< 0.5 ml/kg). She was administered packed cells to maintain the PCV around 40% to a total volume of 1200 ml.

During the latter 24 hours of the critical phase of DF, she had severe metabolic acidosis with lactic acidosis: PH of 7.2, bicarbonate 8 mmol/L, and partial pressure of carbon dioxide 16 mmHg with a lactate level of 12 mmol/L, which was corrected medically with 8.4% sodium bicarbonate 200 ml in divided boluses. Her ionized calcium was persistently low and corrected with multiple boluses of intravenously administered calcium gluconate. Her blood sugar was checked every 2 hours and corrected accordingly.

At the end of presumed critical phase, we gave her 5500 ml of fluid including normal saline, dextran, and packed cells. She was conscious, rational, but drowsy. She had a spiking high temperature. She was icteric but not pale. She was breathless at rest with oxygen saturation of 85% on room air, which increased to 95% with 60% oxygen via mask. Her pulse rate was 120 beats per minute with blood pressure of 140/100 mmHg. She had bilateral pleural effusions up to mid zone. Her liver was 5 cm below the costal margin with normal upper border and markedly tender. She had gross ascites in a horseshoe-shaped distribution. Her serum creatinine was raised to 345 micromol/L with serum potassium of 5.8 mmol/L and during the last 6 hours of the critical phase she was anuric. Her ALT was 8010 U/L and AST 41546 U/L. Her prothrombin time was 22.1 seconds (control 12 seconds) and activated partial thromboplastin time (APTT) was 42 seconds (control 26 seconds). C-reactive protein (CRP) was 240 U/L. Her blood sugars were elevated toward the end of presumed leaking phase of DHF.

At the end of the presumed leaking phase of DHF our patient had: massive liver necrosis; acute kidney injury with acidosis, hyperkalemia, and anuria; deranged clotting with bleeding; and symptomatic volume overload with large plural effusions and gross ascites. Many concerns rose at this point regarding management:

1. The fluid in the third space mainly in the pleural and peritoneal cavities would get reabsorbed and as she probably had an established acute kidney injury with anuria, the reabsorbed fluid would accumulate in her intravascular compartment leading to expansion of intravascular volume and massive volume overload with pulmonary edema and heart failure.
2. Massive liver necrosis with deranged synthetic function would worsen the lactic acidosis which in turn would have a negative effect on the inotropic effect of her heart, clotting derangements might aggravate the bleeding risk, and ongoing hypoxia of the liver might further damage her liver.
3. Sepsis with high fever and elevated inflammatory markers. What is the focus?
4. Management of uncontrolled blood sugar. Is it due to her existing type of diabetes or pancreatitis?

**Fig. 1:**
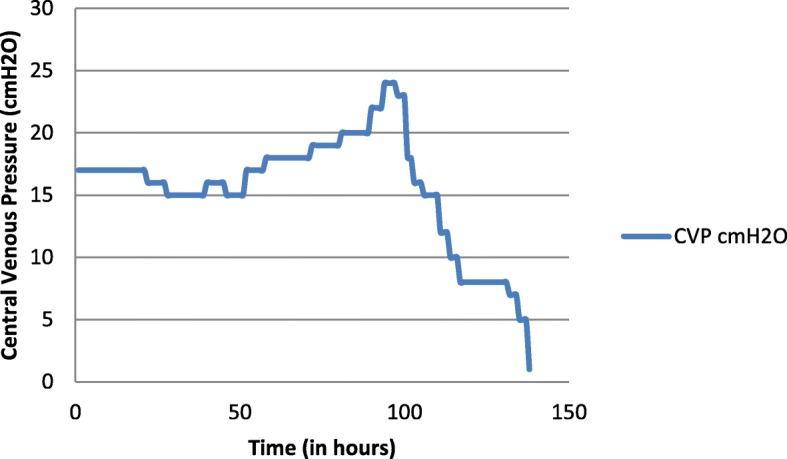
Change in the central venous pressure of the patient over time. *CVP* central venous pressure

**Fig. 2:**
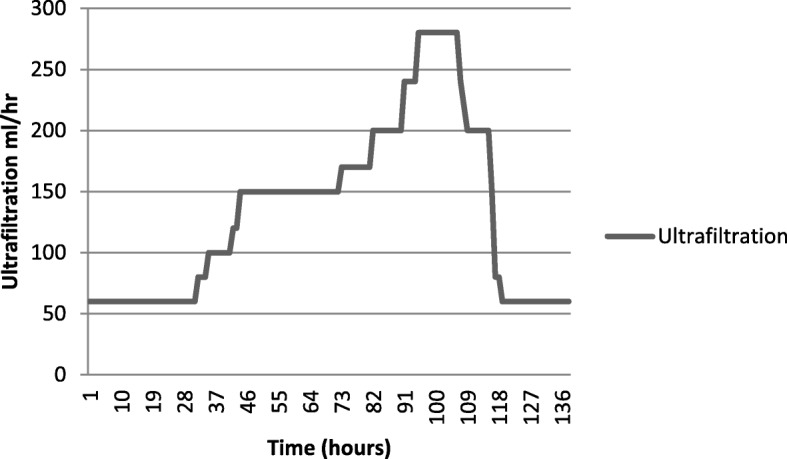
The ultrafiltration removed by continuous veno-venous hemodialysis per hour plotted against time

She was started on continuous renal replacement therapy (CRRT) with CVVHD. We decided to keep CVVHD running and titrate the ultrafiltrate according to the volume state. Observations on central venous pressure (CVP) and blood pressure were made hourly. Intermittent measurements of her inferior vena cava (IVC) diameter and internal jugular vein (IJV) diameter were noted. We assumed that reabsorption of the fluid in the third space would increase the CVP, distend the IJV and IVC, and would increase mainly the diastolic pressure. Depending on the above assumptions, observations were made and ultrafiltrate was gradually increased. Surprisingly, fluid reabsorption occurred in an exponential pattern over a period of 5–6 days and came to a halt abruptly (Figs. 1 and 2). Maximum ultrafiltrate was 280 ml/hour. Heparin was not used in CVVHD due to high risk of bleeding.

She was started on intravenous NAC 100 mg/hour infusion which was continued for 5 days. She was given orally administered metronidazole 400 mg 8 hourly and syrup lactulose to maintain bowel motion 2–3 times per day. She was started on an intravenous infusion of proton pump inhibitors, intravenously administered tranexamic acid, and orally administered norethisterone. She was given intravenously administered vitamin K 10 mg daily for 3 days. She was given 4 units of fresh frozen plasma and 10 units of cryoprecipitate, and 6 units of platelets to correct the coagulopathy. She was transfused with packed cells to maintain PCV around 40% in order to maintain adequate oxygenation of hepatocytes. CVVHD was continued and her lactate level was noted to decline gradually.

She had spiking high fevers on day 3 of hospital stay with high CRP. Septic screening was done with blood culture and urine culture and intravenously administered ceftriaxone was changed over to renal-adjusted dose of intravenously administered meropenem and teicoplanin. Later cultures were negative after 72 hours of incubation. However, gradually her fever settled by lysis of fever over the days.

Although during the presumed critical phase her blood sugar was rather low, her blood sugar started to rise over the days. She was a type 2 diabetic with good control with metformin. Her blood sugar was checked hourly and insulin infusion was continued and titrated according to her blood sugar. Her amylase was 450 U/L (normal range 1–37 U/L).

Over a period of 6 days she was closely monitored. Gradually her transaminases declined, her lactate level normalized, and serum creatinine reduced and normalized (Table 1). Her urine output gradually increased. CVVHD was terminated after 138 hours of dialysis. She was discharged on ninth day of admission after restoring her full physiology. She was discharged on Mixtard insulin (biphasic isophane insulin injection) for diabetic control. She was reviewed in the ward after 3 days, 7 days, and at 1 month after discharge. Her renal functions and liver functions were within the normal range. She was started again on metformin and atorvastatin 1 week after discharge and insulin was discontinued.

**Table 1:** Summary of the basic blood investigations of the patient during hospital stay

Day	1	2	3	4	5	6	7	8	9	10
Hemoglobin (g/dL)	15.3	14.2	11	11.3	12.8	12	11.5	11.8	12	12.5
White cell count (10^6^/microL)	3.22	5.5	7.5	7	6.6	4.6	5	5.4	7	7.5
Platelets (10^3^/microL)	74	21	27	46	79	80	126	146	189	224
Sodium (mmol/L)	146	150	141	136	139	136	135	132	135	135
Potassium (mmol/L)	5.8	4.9	4.2	4.4	4	4.5	3.9	3.2	3.4	4.2
Creatinine (micromole/L)	277	345	432	241	172	160	280	308	150	97
Calcium (ionized)	1.95		1.7							
PT/INR	1.43		1.93	1.98	1.65	1.08		1.1		
ALT (U/L)	6542	8010	4678	3103	1607	932	260	104	100	88
AST (U/L)	30617	41546	24331	5612	1472	302	250	250	110	74
ALP (U/L)	245					156				140
Bilirubin (micromole/L)	48			75.5			68.2			21
GGT (U/L)	N/A									
Albumin (g/L)	25	24	27	29	32		35			
CRP (mg/L)	138	145	150	134	254	208	160		84	46

*ALP* alkaline phosphatase, *ALT* alanine transaminase, *AST* aspartate transaminase, *CRP* C-reactive protein, *GGT* gamma-glutamyltransferase, *N/A* not available, *PT/INR* prothrombin time-international normalized ratio

## Discussion

DF is a common mosquito-borne viral disease among humans seen mainly in the Asia-Pacific region [1]. It can present with a diverse clinical spectrum ranging from asymptomatic infection or simple undifferentiated fever to DHF with multiorgan failure. Four distinct dengue viral serotypes (DEN-1 to DEN-4) are known to cause illness. Infection with one serotype confers protection from reinfection with the same serotype, while reinfection with different serotypes confers no long-term protection and may even predispose plasma leak and worse clinical outcome [2]. No specific antiviral therapy is available for DF. Dengue infection can present with various unusual manifestations. Most of these manifestations of DF are under-reported, under-recognized, or not casually linked to DF including hepatitis and liver failure [3], myositis [4], and encephalitis and other neurological manifestations [5].

We report a case of a middle-age woman with diabetes and dyslipidemia who presented late to our hospital while peaking in the leaking phase of DHF. Her DHF was complicated with massive liver necrosis, acute renal failure with anuria and gross volume overload, and secondary sepsis. After an extensive literature search we believe that this case is the first who survived this sort of complicated DF. We would like to discuss the management principles that we employed in this success story.

Liver dysfunction is a well-recognized feature in both DF and DHF. Liver involvement in dengue infection could be suspected in patients with DF complaining of abdominal pain, nausea, vomiting, and anorexia [6]. Hepatomegaly is present in both DF and DHF but more common in DF [7]. Clinical jaundice has been detected in 1.7–17% of cases in various series [7, 8]. A mild to moderate increase in the transaminases is common in DF and DHF, and AST was higher than ALT [9–11]. The AST released from damaged striated muscle, cardiac muscle, and erythrocytes could explain the levels of AST that are higher than those of ALT in patients with DF at an earlier stage [12, 13]. Therefore, a rise in AST might not be a true reflection of hepatic involvement. The pathogenesis of liver injury in dengue infection is yet to be fully elucidated. Possible hypotheses include direct effects of the virus or host immune response on liver cells, circulatory compromise, and metabolic acidosis and/or hypoxia caused by hypotension or localized vascular leakage inside the liver [14]. Studies have shown that DENV readily infects the liver cells in mouse models [15]. High levels of cytokines particularly interleukin-22 (IL-22) and interleukin-17 (IL-17) were found in mouse models which may be responsible for the cytokine-induced liver damage [16]. Sung *et al.* observed the infiltration of hepatocytes with natural killer cells followed by T cells and this was found to be associated with the apoptosis of hepatocytes [17].

Histopathological studies of postmortem specimens of patients who had a fatal outcome have shown that the liver is congested with liver cell necrosis and apoptosis, predominantly in midzonal and centrilobular areas, macrovascular steatosis, and Councilman bodies. Many postmortem reports show little or no inflammation [18, 19]. It is interesting that similar centrilobular necrosis is a typical finding in hypoxic hepatitis [20]. Considering the fact that a severe form of liver necrosis is seen among the patients with DHF who present late with prolonged shock, we can postulate the fact that hypoxic injury due to reduced hepatic perfusion is probably an important contributor to the causation of liver damage. By contrast, few cases of fulminant liver failure have been reported in the absence of shock [3]. Khongphatthanayothin *et al.* reported an interesting case of liver failure from DENV infection with reversal of portal venous blood flow [21]. They postulated that hepatic sinusoidal obstruction coupled with shock might have been the underlying mechanism of liver failure in this disease [21].

Our patient presented at the peak of the leaking phase and collapsed on admission with cold and clammy peripheries. She had very high transaminases with deranged clotting and venous blood gas showing elevated lactate levels. Her liver involvement could be multifactorial including direct viral damage, immunological damage, hypoxia due to dehydration, and intravascular volume depletion due to leaking. It was further exacerbated by ongoing gastrointestinal and per vaginal bleeding. We aggressively resuscitated with crystalloids followed up by colloids. We considered transfusion of packed cells to maintain PCV around 40% to maintain adequate oxygenation to her liver.

She was started on intravenous NAC infusion at a rate of 100 mg/hour. NAC scavenges free radicals, improves antioxidant defense, and acts as a vasodilator to improve oxygen delivery and consumption [22]. However, limited data are available in the literature regarding the efficacy of NAC in DF-related liver dysfunction. A retrospective analysis on NAC in dengue-associated liver failure by Kumarasena *et al*. showed that five patients who survived out of eight were in early (coma grade 1, 11) liver failure stage at the time when NAC was started [23]. Habaragamuwa and Dissanayaka reported another case of hepatitis following dengue treated with NAC with success [24]. Large randomized trials should be carried out to establish its efficacy along with appropriate dosage, timing, and duration of treatment. We decided to continue NAC until our patient’s liver enzymes were less than 500 U/L.

Lactic acidosis resulting from excess accumulation of lactate and protons is associated with increased mortality and poor clinical outcome [25]. Hyperlactatemia occurs when lactate production exceeds lactate consumption. In tissue hypoxia lactate is overproduced as a result of decreased mitochondrial oxidation [26] that could be either due to generalized hypoxia or microcirculatory dysfunction [27, 28]. Coexisting acidosis might further reduce the renal excretion of lactate. At the hyperdynamic stage of sepsis or shock, epinephrine-dependent stimulation of the β_2_-adrenoceptor augments the glycolytic flux both directly and through enhancement of the sarcolemmal Na^+^, K^+^-ATPase which lead to overproduction of lactate [29]. The liver accounts for up to 70% of whole-body lactate clearance [30]. Hyperlactatemia is common in acute fulminant liver disease, reflecting both reduced clearance and increased production of lactate by the liver [31]. Metformin interferes with oxidative phosphorylation and suppresses hepatic gluconeogenesis which can lead to hyperlactatemia in rare cases [32].

The lactic acidosis that developed in our patient could be multifactorial. She had poorly controlled diabetes and was on the maximum dose of metformin, she presented in severe shock due to plasma leakage and dehydration, and developed acute liver failure and renal failure. All these events could have contributed to her hyperlactatemia. High lactate levels will further suppress myocardial contractility and worsen the acidosis which will act as a vicious cycle deteriorating physiology.

The management strategies employed in this patient were:

1. Aggressive resuscitation with fluids during the initial shock including crystalloids and colloids.
2. Use of packed cells to maintain PCV around 40% to improve oxygen carrying capacity.
3. Supplementary oxygen to the patient during shock.
4. Use of CVVHD employed as a form of renal replacement therapy to remove lactate from the body and correct the acidosis.

Patients with DHF develop selective plasma leakage manifested as accumulation of fluid in pleural and abdominal cavities and hemoconcentration. The leakage is assumed to last approximately 48 hours and is followed by a spontaneous and rapid resolution but has wide individual variations [33]. Increased vascular permeability is mediated by an interplay between DENV, immune cells and endothelial cells with adhesion molecules, enzymes and cytokines according to the current evidence [33]. Very limited information is available in the literature regarding the rate and duration of plasma leak and about reabsorption of fluid in the pleura and peritoneal cavities during recovery. Understanding the trends in fluid leakage and reabsorption of dengue has been hampered by a lack of animal models.

During recovery from DHF, extravasated fluid will be reabsorbed and a functioning kidney would filter the extra volumes of fluid and the patient will be polyuric. Our patient’s clinical course was complicated by acute renal failure, which was probably the result of acute tubular necrosis due to her late presentation with prolonged shock. She was anuric in the initial days of recovery. She was given 5500 ml of fluid during the presumed leaking phase of DHF and she had bilateral moderate to severe pleural effusions and gross ascites. We hypothesized that if she reabsorbed this excess fluid in the third space into the intravascular compartment with non-functioning renal tissue to filter it out, she would soon be intravascularly expanded which would lead to acute left ventricular failure and pulmonary edema. So we decided to have a renal replacement method prophylactically anticipating the reabsorption of fluid in the cavities. CVVHD was selected as the method of renal replacement therapy as it can be used continuously for a longer period, with less effect on the hemodynamics as opposed to intermittent hemodialysis.

What is the guide to assess the rate and volume of fluid reabsorption into the intravascular compartment?

We decided to make serial measurements of

1. CVP
2. IVC diameter and collapsibility
3. IJV diameter and collapsibility
4. Diastolic blood pressure (DBP)

CVP and DBP were considered as hard measurements, whereas IJV and ICV diameters and collapsibility had intra-operator and inter-operator variability. Ultrafiltration of the CVVHD was titrated according to the above measurements. Interesting observations were made during this period.

Our patient’s CVP gradually increased reflecting reabsorption of fluid into the vascular compartment and we gradually increased the ultrafiltration starting from a figure of 60 ml/hour. CVP peaked in the 95th hour of CVVHD and ultrafiltration was increased in an exponential manner up to 280 ml/hour. CVP was maintained at the peak for 4 hours and a dramatic decline was noted in the central pressures indicating termination of fluid reabsorption. Over the next 24 hours CVP dropped from 24 cm H_2_O to 1 cm H_2_O and ultrafiltration was brought down to zero rapidly and CVVHD terminated. During the period of dialysis she was maintained on orally administered fluids at 50 ml per hour and she was anuric on initial 3 days of CVVHD and gradually improved over time. Total ultrafiltration removed 18.5 L over a period of 138 hours. She would not have survived the exponential rise in intravascular volume during reabsorption with poor renal function. The key for survival was the timely arrangement of renal replacement therapy.

Fluid dynamics during reabsorption has never been described before in DHF. In the index patient reabsorption took a longer period than is described in a timeframe of 48 hours in the literature and it occurred in an exponential pattern, plateaued, and abruptly ceased.

## Conclusions

Management of a patient with DHF complicated with acute liver failure, lactic acidosis, and acute kidney injury is challenging. Although conclusions cannot be drawn from a single case report we emphasize importance of the following management strategies.

1. Use of NAC in hepatic dysfunction in dengue illness.
2. Increasing PCV by transfusing blood to increase the oxygen-carrying capacity even in the absence of a compelling indication for a transfusion in the “standard care.”
3. Lactate level to be used as a prognostic marker and as a tool to guide treatment.
4. Use of CVVHD in those with acute renal impairment and DHF during the recovery phase to maintain intravascular volume without leading to heart failure and pulmonary edema.

Large randomized trials should be carried out to establish the efficacy of these treatment strategies to support the above observations and change current practice.

We believe that the fluid dynamics we have observed in the index case need to be tested in a proposed animal model and it will open up new research areas and will generate new knowledge in the management of DF.
